# Antibacterial Activity of *Mulinum spinosum* Extracts against Slime-Producing *Staphylococcus aureus* and Methicillin-Resistant *Staphylococcus aureus* Isolated from Nasal Carriers

**DOI:** 10.1155/2014/342143

**Published:** 2014-11-03

**Authors:** Echenique Daniela, Chiaramello Alejandra, Rossomando Pedro, Mattana Claudia, Alcaráz Lucía, Tonn Carlos, Laciar Analía, Satorres Sara

**Affiliations:** ^1^Department of Biochemistry and Biological Sciences, National University of San Luis, 5700 San Luis, Argentina; ^2^Department of Chemistry, National University of San Luis, 5700 San Luis, Argentina

## Abstract

Nasal carriers of *Staphylococcus aureus* are important reservoirs with risk of developing endogenous infections or transmitting infections to susceptible individuals. Methicillin-resistant *S. aureus* (MRSA) are associated with higher rates of treatment failure. Some strains of *S. aureus* produce slime which is believed to make the microorganisms more resistant to antibiotics and host defenses. The antibacterial activity of ethyl acetate : n-hexane (EtOAc : HEX) extracts of *Mulinum spinosum* (5 : 95% EtOAc : HEX, 50 : 50% EtOAc : HEX, 70 : 30% EtOAc : HEX and mix 20 : 80/30 : 70% EtOAc : HEX, 50 : 50/70 : 30/100 : 0% EtOAc : HEX) were assayed against 3 slime-producing *S. aureus* strains and 2 MRSA strains isolated from nasal carriers. *S. aureus* ATCC 35556 slime-producing strain and MRSA ATCC 43300 strain were used as controls. The extracts were prepared using flash chromatography. *M. spinosum* 5 : 95% AcOEt : HEX showed antibacterial effect against all slime-producing strains (MIC: 500 *µ*g/mL) and the highest activity against MRSA strains (MIC: 500 to 1000 *µ*g/mL). All *M. spinosum* extracts assayed were active against slime-producing *S. aureus* and MRSA at doses between 500 and 4000 *µ*g/mL. Both, slime-producing *S. aureus* and MRSA are highly contagious and hardly eradicated by antibiotic therapies. So, there is an increasing need to find new substances with the ability to inhibit these strains.

## 1. Introduction

*S. aureus* is widely distributed in nature and is part of the bacterial human flora of the skin, armpits, groin, perineum, upper respiratory tract, and anterior nares [1]. In healthy adults the 20% are persistent nasal carriers of this bacterium [2]. So, carriers of* S. aureus* are important reservoirs with risk of developing endogenous infections or of transmitting infections to susceptible individuals. Certain strains of* S. aureus* produce slime and this exopolysaccharide is a mucoid material, which is firmly attached to the bacterial cell wall and is released to the environment [3]. Slime production has been implicated as a virulence factor and is postulated to be a mechanism by which bacteria attach to and colonize indwelling medical devices [4]. The importance of the role played by slime is further increased by its frequent association to reduced antibiotic susceptibility [5]. The presence of these microbial communities is often associated with various chronic diseases including cystic fibrosis, periodontitis, chronic prostatitis, otitis media, endocarditis, and recurrent urinary tract infections [6]. Methicillin-resistant* S. aureus* (MRSA) are associated with higher rates of treatment failure by the limited availability of antibiotics showing activity* in vivo*. The main impact of this microorganism is that MRSA strains were traditionally limited to the hospital environment and today have become important pathogens of the community [7, 8]. Due to increased resistance to antibiotics there is an imminent need to search for new therapeutic options [9, 10].

Ethnobotany is the main source for development and research of natural drugs and has received considerable interest in recent years. Latin American countries have a rich tradition in the use of medicinal plants in folk medicine [11].* Mulinum* is a genus of herbaceous plants belonging to the Apiaceae family. It comprises 37 described species. The type species of this genus is* Mulinum spinosum* Pers.* M. spinosum* (neneo, hierba negra) is a plant endemic of the mountains of Chile and western of Argentina Patagonia region. It is a thorny and perennial shrub [12]. This species is used as an analgesic for the treatment of dental neuralgias, in the hepatic and urinary diseases and altitude sickness [13, 14]. Moreover, in the field, it is used as medicinal plant against toothache [15].

The aim of this work was to study the inhibitory activity of* M. spinosum* extracts against slime-producing* S. aureus* and MRSA isolated from nasal carriers.

## 2. Materials and Methods

### 2.1. Plant Material

*M. spinosum* (Cav.) Pers was collected in the Cordillera de Los Andes, Uspallata, Mendoza, Argentina. Voucher specimen was identified by Ing. Del Vitto et al. and lodged in the University of San Luis, Argentina, herbarium (N°9092) [16].

### 2.2. Preparation of Extracts

Previously dried aerial parts at room temperature and finely powdered were macerated with acetone at room temperature for 48 h. Acetone extract was separated by filtration. Extraction was replicated 3 times. Extraction fluids were concentrated under reduced pressure yielding 330 g of dark syrup and then it was dissolved with acetone and absorbed on silica gel column. Each acetone extract was partitioned by chromatography “flash” using us elution solvents mixtures of ethyl acetate and n-hexane (EtOAc/HEX) of increasing polarity. The progress of separation was monitored by thin layer chromatography (TLC) using as mobile phase benzene : dioxane : acetic acid (120 : 20 : 4) and as revealing a mixture of sulfuric acid : acetic acid : H_2_O (4 : 20 : 1) followed by heating at 120°C [17].

In this study, we evaluated* in vitro* the antibacterial activity of 5 : 95% EtOAc : HEX, 50 : 50% EtOAc : HEX, 70 : 30% EtOAc : HEX and mix 20 : 80/30 : 70%, EtOAc : HEX 50 : 50/70 : 30/100 : 0% EtOAc : HEX extracts of* M. spinosum*.

### 2.3. Microorganisms

A total of 24* S. aureus* strains isolated from nasal carriers, kept in the ceparium (maintained in the culture collection) of the Laboratory of Microbiology of the National University of San Luis, were assayed for slime production and oxacillin resistance. Then, the antibacterial activity was assayed against a total of 5 of those strains: three slime-producing* S. aureus* and 2 MRSA isolated from nasal carriers.* S. aureus* ATCC 35556 slime-producing strain and MRSA ATCC 43300 strain were used as controls.

### 2.4. Slime Production

Slime production was performed by using 2 methods as follows.

#### 2.4.1. Congo Red Agar Method

It was performed according to Freeman et al. [18] with the following modifications: the strains were streaked onto Congo red agar (CRA) plates (0.8 g of Congo red and 50 g of sucrose in 1 liter of brain heart infusion agar), incubated for 24 h at 37°C and subsequently overnight at room temperature. Plates were inspected for the color of the colonies at 24 h. For colonies color evaluation, a four-color reference scale was used: black and bordeaux almost black as slime-producing strains and bordeaux and red as nonslime-producing strains.

#### 2.4.2. PCR Method for the Amplification of the* ica*A and* ica*D

PCR reactions were performed using the method described by Arciola et al. [19]. In brief, 2 pairs of primers were designed for the detection of* ica*A. The following primers were used: 5′-ACAGTCGCTACGAAAAGAAA as the forward primer and 5′-GGAAATGCCATAATGACAAC as the reverse primer, yielding a PCR product of 103 bp. For the detection of* ica*D the following primers were used: 5′-ATGGTCAAGCCCAGACAGAG and 5′-CGTGTTTTCAACATTTAATGCAA as forward and reverse primers, respectively, yielding a PCR product of 198 bp. DNA amplification was carried out with the following thermal cycling profile: initial denaturation at 94°C for 5 min, followed by 50 cycles of amplification (denaturation at 94°C for 30 s, annealing at 59°C for 30 s, and extension at 72°C for 30 s) with a final extension at 72°C for 1 min. After the first 30 cycles, a further 1 U of Taq DNA polymerase was added. PCR products were analyzed by electrophoresis in 2% agarose gel for 50 min at 80 V. The bands were stained with GelRed and observed under UV light.

### 2.5. Oxacillin Resistance

*S. aureus* isolates were screened for oxacillin resistance using disk diffusion method [20].

### 2.6. Antibacterial Activity

#### 2.6.1. Determination of Minimal Inhibitory Concentration (MIC)

The antibacterial activity was assayed* in vitro* using microplate method (microwell dilution) according to the CLSI method [21] in tripticase soya broth (Britania, Argentina) pH 7,2 supplemented with 0,01% (w/v) of 2,3,5-triphenyltetrazolium chloride (TTC) used as visual indicator of bacterial growth. The inoculum of each strain was prepared from 24 h broth culture and adjusted to concentration of 10^6^ CFU/mL. Organic extracts were dissolved in dimethylsulfoxide (DMSO) and tested in a concentration ranging from 8 to 0.1 mg/mL. The 96-well plates were prepared by dispensing into each well 95 *μ*L of nutrient broth and 5 *μ*L of the inoculum (final concentration of 10^4^ CFU/mL). One hundred microliter aliquots from the serial dilutions of extracts were transferred into 4 consecutive wells. The final volume in each well was 200 *μ*L. Controls of nutrient broth, strains, DMSO, and extracts were included. After 24 h incubation at 37°C, the antibacterial activity of the extracts (MIC) was defined as the lowest concentration of the extract in the medium in which there is no visible grown. The experiments were replicated at least twice.

#### 2.6.2. Determination of Minimal Bactericidal Concentration (MBC)

Extracts that showed inhibitory activity in the preliminary broth assay were submitted to a subculture on the surface of the tripticase soya agar plates, in order to evaluate bactericidal effect. The presence or absence of bacterial growth was determined by visual inspection. MBC was defined as the lowest concentration that showed no bacterial growth in the subcultures after 24 h of aerobic incubation at 37°C.

## 3. Results and Discussion

**Figure 1:**
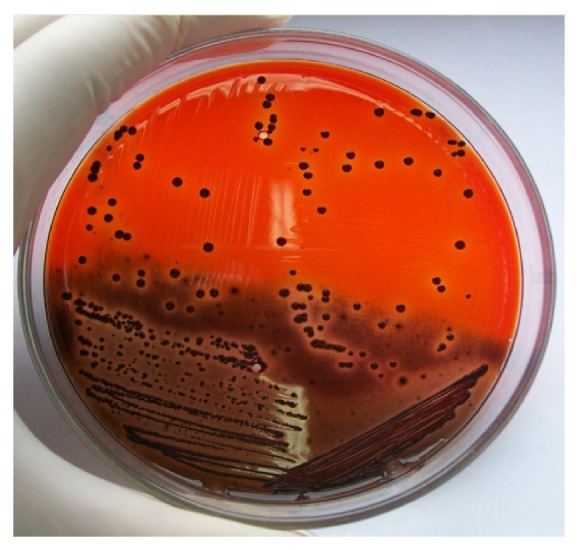
Culture on CRA: black colonies of slime-producing* S. aureus*.

**Figure 2:**
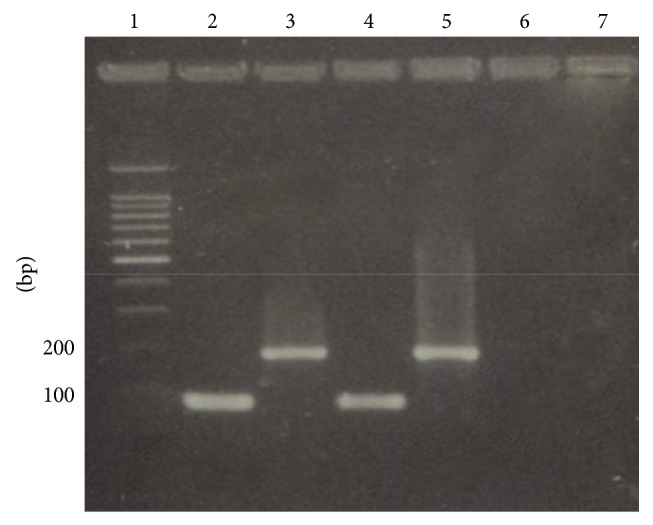
PCR detection of* ica*A and* ica*D genes. Lane 1: molecular size marker; lane 2:* S. aureus *ATCC 35556 (*ica*A); lane 3:* S. aureus *ATCC 35556 (*ica*D); lane 4: slime-producing* S. aureus *(*ica*A); lane 5: slime-producing* S. aureus *strain (*ica*D); and lanes 6 and 7:* S. epidermidis *ATCC 12228 (*ica*A and* ica*D, resp.).

Of the 24* S. aureus* strains studied, 3 were slime positive by both methods assayed and 2 showed oxacillin resistance. The slime-production by cultures on CRA is observed in Figure 1:* S. aureus* appear as black colonies.  Figure 2 shows PCR method: 103-bp band for* ica*A gen and 198-bp band* ica*D gen obtained with DNA from slime-producing* S. aureus*.

Extract of* M. spinosum* 5 : 95% AcOEt : HEX showed antibacterial effect against all slime-producing strains of* S. aureus* isolated from nasal carriers (MIC: 500 *μ*g/mL).* M. spinosum* 50 : 50% AcOEt : HEX and mix* M. spinosum* 20 : 80/30 : 70% AcOEt : HEX extracts showed inhibitory activity against slime-producing strains isolated from nasal carriers with MIC between 500 *μ*g/mL and 2000 *μ*g/mL.* S. aureus* ATCC 35556 was sensitive to these 3 extracts at doses 1000 *μ*g/mL. The* M. spinosum* 70 : 30% extract and 50 : 50/70 : 30/100 : 0% EtOAc : HEX mix showed the lowest antibacterial activity, MICs between 2000 *μ*g/mL and 4000 *μ*g/mL.* S. aureus* ATCC 35556 was inhibited with MIC = 4000 *μ*g/mL. Higher concentrations (one to three times higher than the corresponding MICs values) of extracts were needed to obtain bactericidal effect. Only the extracts* M. spinosum* 70 : 30% AcOEt : HEX and* M. spinosum* 20 : 80/30 : 70% AcOEt : HEX showed the same MIC and MBC values for* S. aureus* ATCC 35556 (Table 1).

**Figure 3:**
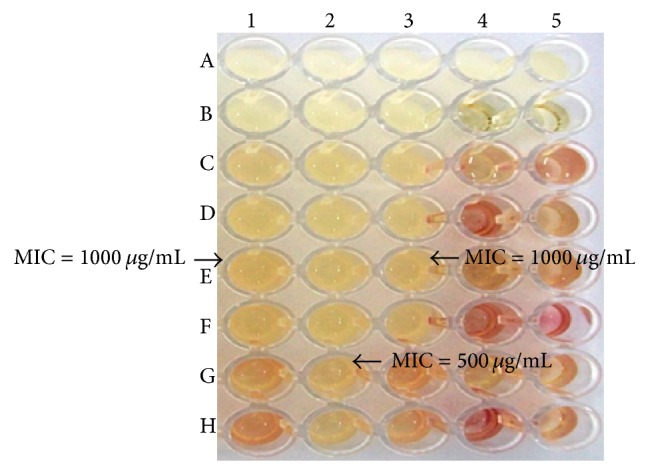
Microwell dilution in broth. Extract:* M. spinosum *5 : 95% AcOEt : HEX. 1:* S. aureus *ATCC 43300; 2 and 3: MRSA strains isolated from nasal carriers; 4 and 5 (A): extract control; 4 and 5 (B): broth controls; 4 and 5 (C–E): DMSO controls; and 4 and 5 (F–H): strains controls.

In addition, our study on the activity of the extracts against MRSA strains isolated from nasal carriers showed that* M. spinosum* 5 : 95% AcOEt : HEX extract presented the highest activity (MIC: 500 and 1000 *μ*g/mL).* M. spinosum* 50 : 50% AcOEt : HEX and 20 : 80/30 : 70% extracts showed activity against MRSA (MIC: 2000 *μ*g/mL). Extracts of* M. spinosum* 70 : 30% and 50 : 50/70 : 30/100 : 0% AcOEt : HEX inhibited the growth of MRSA at doses of 4000 *μ*g/mL.* S. aureus* ATCC 43300 was inhibited by all extracts (MIC between 500 *μ*g/mL and 4000 *μ*g/mL). The MBC values were one- or twofold higher than the corresponding MIC values, except for extracts* M. spinosum* 5 : 95% AcOEt : HEX and* M. spinosum* 70 : 30% AcOEt : HEX extracts that showed the same MIC and MBC values for* S. aureus* ATCC 43300 (Figure 3 and Table 2).

To our knowledge, there are few reports available in the literature on the antistaphylococcal activity of* M. spinosum *extracts. However, organic extracts of other plants show activity against those bacteria. For example, Marino et al. evaluated* in vitro* effect of branch methanol and aqueous extracts of five* juniperus* species on the growth, adherence, and biofilm formation of* S. aureus. *All the extracts affected the biofilm development depending on the biofilm-forming strain capacity and, in our study, inhibited bacterial growth of all assayed strains [22].

Moreover, Saising et al. [23] demonstrated that* Rhodomyrtus tomentosa* ethanol extract and its pure compound rhodomyrtone both possessed strong activity against biofilm-forming staphylococci isolated from acne lesions with MICs of 32–128 *μ*g/mL and 0,5–1 *μ*g/mL, respectively. However, the MIC of this extract was lower than the MIC of all extracts assayed in our study.

Moreover, Quave et al. [24] studied extracts of wild plants grown in southern Italy for the inhibition of growth and biofilm formation in MRSA. These authors demonstrated that ethanolic and water extracts had limited bacteriostatic activity, and, in fact, many promoted planktonic growth. In contrast, our results show that all the tested extracts showed good activity against MRSA.

About the chemical composition of* M. spinosum*, some authors investigated families of secondary metabolites in leaves, flowers, and fruits. Three group compounds (saponins, flavonoids, and terpenoids/sterols) were identified in fruits and flowers but they were absented in leaves [25–27]. Terpenoids, glycosteroids, flavonoids, and polyphenols are small molecules naturally produced by plants that can inhibit many bacterial species, particularly gram-positive organisms. These compounds are receiving sustained attention regarding their potential use since there has been strong evidence that they possess, in addition to antimicrobial activity, anti-inflammatory and antitumour properties [22, 28–30].

Schito et al. [28] showed that diterpenoid compounds obtained from the exudate produced by the aerial parts of* Salvia corrugata *inhibited the synthesis of biofilm* in vitro* produced by multiresistant* S. aureus*,* S. epidermidis*, and* Enterococcus faecalis*. These compounds presented MICsof 3200–6400 *μ*g/mL when they were assayed against* S. aureus*. Also, these investigators indicate that such diterpenoids were active against all strains of methicillin-resistant* S. aureus* tested.

Also, some studies have demonstrated in plants belonging to* Mulinum* genus an interesting group of bioactive metabolites, such as mulinane diterpenoids, and Molina-Salinas et al. evaluated the antituberculosis activity of natural and semisynthetic mulinane diterpenoids isolated from* M. crassifolium* which showed activity against* Mycobacterium tuberculosis* strains [31].

Previous studies of our research group have isolated two new diterpenes from* M. spinosum* acetone extracts: 14-*α*-hydroximulinolic and mulin-12-ene-14-one-20-oic acids. Other compounds, such as mulinolic acid and 11,13-dien-20-oico mulin acid, were identified from the fraction 10% AcOEt/HEX obtained by* M. spinosum*, too [27]. Probably some of these metabolites are responsible for the antistaphylococcal activity observed in this work.

It is known that slime-producing strains are highly resistant to antibiotics, being able to survive against antibiotic concentrations thousands of times greater than in planktonic bacteria [32]. Moreover, MRSA presents a significant threat to public health, infection has reached epidemic proportions, and therapeutic options are limited because these strains are often resistant to a lot of antimicrobial agents [33, 34]. The present study showed that all the extracts of* M. spinosum *assayed were able to inhibit strains of* S. aureus* which presented these virulence factors at doses between 500 *μ*g/mL and 4000 *μ*g/mL.

## 4. Conclusion

Both, slime-producing* S. aureus* and MRSA present a significant dilemma to medicine today, are highly contagious, and hardly eradicated by antibiotic therapies. So, there is an increasing need to find new compounds with the ability to inhibit these strains. Extracts of* M. spinosum* were active against all strains of slime-producing* S. aureus* and MRSA assayed, so extracts of this plant could represent interesting sources of natural antibiotics and justify the realization of further studies about the antimicrobial activity and characterization of new active compounds.

## Figures and Tables

**Table 1:** Minimal inhibitory concentration and minimal bactericidal concentration of *M. spinosum* extracts against slime-producing *S. aureus*.

*Mulinum spinosum* ethyl acetate/n-hexane extracts					
MIC/MBC (*μ*g/mL)					
Slime-producing strains	5 : 95%	50 : 50%	70 : 30%	20 : 80/30 : 70%	50 : 50/70 : 30/100 : 0%
*S. aureus* NC1	500/1000	500/1000	2000/4000	500/1000	2000/4000
*S. aureus* NC2	500/1000	500/1000	4000/8000	500/1000	2000/4000
*S. aureus* NC3	500/1000	1000/2000	4000/8000	2000/1000	2000/4000
*S. aureus* ATCC 35556	1000/2000	1000/2000	4000/4000	1000/1000	4000/8000

NC: nasal carrier.

**Table 2:** Minimal inhibitory concentration and minimal bactericidal concentration of *M. spinosum* extracts against methicillin-resistant *S. aureus*.

*Mulinum spinosum* ethyl acetate/n-hexane extracts					
MIC/MBC (*μ*g/mL)					
MRSA	5 : 95%	50 : 50%	70 : 30%	20 : 80/30 : 70%	50 : 50/70 : 30/100 : 0%
*S. aureus* NC4	500/1000	2000/4000	4000/8000	2000/4000	4000/8000
*S. aureus* NC5	1000/2000	2000/4000	4000/8000	2000/4000	4000/8000
*S. aureus* ATCC 43300	1000/1000	2000/4000	4000/4000	500/1000	4000/8000

NC: nasal carrier.
